# Peptide cargo tunes a network of correlated motions in human leucocyte antigens

**DOI:** 10.1111/febs.15278

**Published:** 2020-03-26

**Authors:** Jade R. Hopkins, Rory M. Crean, Dragana A. M. Catici, Andrew K. Sewell, Vickery L. Arcus, Marc W. Van der Kamp, David K. Cole, Christopher R. Pudney

**Affiliations:** ^1^ Division of Infection and Immunity School of Medicine Cardiff University UK; ^2^ Department of Biology and Biochemistry University of Bath UK; ^3^ Doctoral Training Centre in Sustainable Chemical Technologies University of Bath UK; ^4^ School of Science Faculty of Science and Engineering University of Waikato Hamilton New Zealand; ^5^ School of Biochemistry University of Bristol UK; ^6^ Centre for Therapeutic Innovation University of Bath UK; ^7^ Present address: Immunocore Ltd. Abingdon UK

**Keywords:** allostery, molecular dynamics, peptide–human leucocyte antigen, protein flexibility, T‐cell receptor

## Abstract

Most biomolecular interactions are typically thought to increase the (local) rigidity of a complex, for example, in drug‐target binding. However, detailed analysis of specific biomolecular complexes can reveal a more subtle interplay between binding and rigidity. Here, we focussed on the human leucocyte antigen (HLA), which plays a crucial role in the adaptive immune system by presenting peptides for recognition by the αβ T‐cell receptor (TCR). The role that the peptide plays in tuning HLA flexibility during TCR recognition is potentially crucial in determining the functional outcome of an immune response, with obvious relevance to the growing list of immunotherapies that target the T‐cell compartment. We have applied high‐pressure/temperature perturbation experiments, combined with molecular dynamics simulations, to explore the drivers that affect molecular flexibility for a series of different peptide–HLA complexes. We find that different peptide sequences affect peptide–HLA flexibility in different ways, with the peptide cargo tuning a network of correlated motions throughout the pHLA complex, including in areas remote from the peptide‐binding interface, in a manner that could influence T‐cell antigen discrimination.

## Graphical abstract

TCRs recognise peptide–HLA complexes mediating immune reactivity against pathogens and neoplasms. We investigated the drivers that govern molecular flexibility of different peptide–HLAs. We found that HLA motions were mediated by small alterations in the bound peptide, suggesting that allosteric mechanisms mediate HLA flexibility (‘the tail wags the dog’). Our study sheds new light on the molecular mechanisms guiding protein dynamics in peptide–HLA, the major target for the TCR.

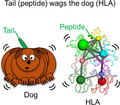

**Abbreviations**
APLs: altered peptide ligands
CAN: community network analysis
DCCMs: dynamic cross‐correlation matrices
ER: endoplasmic reticulum
FEL: free energy landscape
HLA: Human leucocyte antigen
MD: molecular dynamics
*p*/*T*
: pressure/temperature
pHLAs: peptide–human leucocyte antigens
RMSFs: root mean square fluctuations
TCR: T‐cell receptor
T_m_
: melting temperatures

## Introduction

The T‐cell receptor (TCR), expressed on the surface of T cells, scans for antigens on the surface of virtually every cell in the body. TCR–antigen recognition can mediate clearance of germs and neoplasms, and plays a major role in autoimmunity and transplantation [1, 2, 3, 4, 5]. As such, a better understanding of the molecular determinants that govern TCR–antigen interactions is key to identifying novel therapeutic interventions that can enhance (cancer immunotherapy, vaccines) or inhibit (regulation of autoimmunity) T‐cell activation. The natural TCR ligands are the peptide–human leucocyte antigens (pHLA) class I and class II. Classically, pHLA class I is recognised by CD8+ T cells, and pHLA class II is recognised by CD4+ T cells. These ligands feature a number of unique characteristics (analogous in both the pHLA class I and pHLA class II systems) that have important implications for both protein dynamics and T‐cell‐mediated immunity. First, the antigen‐binding site is composed of a composite that includes the HLA‐binding groove (formed by the HLA α1 and α2 domains for HLA class I, the focus from here on in) and a short 9‐ to 13‐amino acid peptide that can be derived from a completely unrelated protein (the source of these peptides is generally the immune proteasome that degrades the majority of intracellular proteins, which can derive from foreign or mutated self‐proteins) [6, 7]. Intriguingly for HLA class I, although the peptide only accounts for ~ 2% of total amino acids in the pHLA class I molecule, its position within the binding groove ‘pins’ the entire complex together, that is HLA class I molecules do not generally form a stable structure without a bound peptide [8]. These peptides are edited by the antigen‐processing machinery in the endoplasmic reticulum (ER) before being transported to the cell surface for TCR interrogation [9]. Second, during binding, the TCR interacts directly with both the HLA surface and the peptide (composite antigen‐binding site) [10, 11]. How the TCR retains the delicate balance between HLA binding and peptide dependence (peptide‐independent recognition of HLA would result in T‐cell activation against virtually every nucleated cell in the body) is still not fully understood. Finally, pHLA is unique in biology because it can form a trimeric complex with both the TCR and co‐receptor molecules (CD8 for pHLA class I and CD4 for pHLA class II) [12, 13, 14, 15]. Although the co‐receptors bind to an invariant site distal from the TCR, this interaction is known to play a role in TCR thymic selection [16], and can tune TCR cross‐reactivity by altering T‐cell potency [17, 18].

Many studies have focused on understanding the relationship between the biophysical characteristics of the TCR‐pHLA interaction and T‐cell potency [19, 20, 21, 22, 23, 24], and the role of TCR flexibility during pHLA engagement [4, 25, 26, 27, 28, 29, 30, 31, 32, 33, 34]. These studies have demonstrated that the optimal TCR‐pHLA interactions can be mediated by a highly flexible binding mode, probably contributing to the ability of TCRs to recognise multiple different pHLAs [19, 35, 36, 37, 38, 39]. This flexibility has been observed in the flexible loops that form the binding site of the TCR, contributing towards the notion that TCRs ‘meld’ around the pHLA surface during binding [40]. Although flexibility has also been reported in both the HLA‐bound peptide [41, 42, 43, 44, 45] and the HLA helices [46, 47, 48], the role that different peptides play in modulating HLA dynamics globally, and what impact the dynamics might have on T‐cell antigen recognition, is only beginning to be explored [49, 50]. On the one hand, a more dynamic pHLA molecule could enable TCR binding of the peptide cargo in an ‘optimal’ conformation for T‐cell activation, or to enable recognition by a greater range of different TCRs. On the other hand, a more dynamic pHLA may confer a higher entropic cost during TCR binding that might reduce affinity, or could lead to the unwanted recognition of self‐antigens leading to autoimmunity.

In the context of protein–protein and protein–ligand interactions, molecular flexibility is defined by a multidimensional free energy landscape (FEL), comprising a large number of energetic minima and maxima that define differently stable conformational substates of the same protein (or protein complex). Peptide‐dependent effects on HLA dynamics could influence the functional interaction between TCR and pHLA, as well as other molecules known to interact with these receptors. As suggested by others, we consider whether the nature of the peptide cargo can alter the conformational states that are accessible to the HLA molecule (i.e. its FEL). To address this hypothesis, we use combined pressure/temperature (*p*/*T*)‐dependent fluorescence spectroscopy and molecular dynamics (MD) simulations to expose differences in the thermodynamics of the differing pHLA complexes and to identify the atomistic determinants of changes in pHLA flexibility. These data provide further insights into the role that the peptide plays in tuning the flexibility of HLA, a feature that might contribute to modulation of TCR–antigen recognition and T‐cell‐mediated immunity.

## Results and Discussion

### Pressure/temperature matrices expose differing thermodynamic contributions to pHLA flexibility

**Fig. 1:**
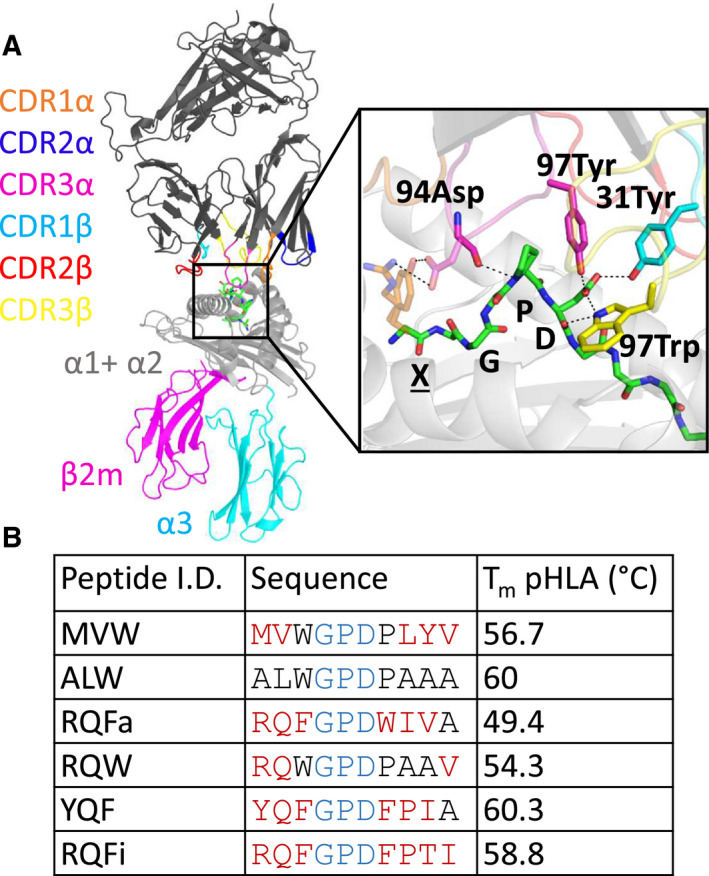
(A) Structural representation of the 1E6 TCR‐pHLA interaction with the inset showing a zoom‐in on the TCR‐pHLA binding site. The conserved GPD motif in the peptide and TCR residues that interact with these residues are shown as sticks. For the 4 peptides that also interact with the TCR via their position 1 residue (X), the side chain of this residue is also indicated with orange sticks. Images were generated with PyMOL 1.8.6 [88]. (B) Peptide sequences and their respective pHLA melting temperatures (*T*_m_) as determined by CD spectroscopy (reported previously [19]). The central conserved GDP motif is coloured blue, and the altered peptide residues with respect to the index ALWGPDPAAA peptide are shown in red.

We focused on the well‐characterised 1E6 TCR system, a TCR that naturally recognises the HLA‐A*02:01‐restricted ALWGPDPAAA_15‐24_ peptide from the preproinsulin protein, and plays a biological role in human type 1 diabetes [2, 51, 52, 53]. We have previously reported a number of altered peptide ligands (APLs) for the 1E6 TCR using structural, biophysical and cellular analysis. These data demonstrated that, despite a highly conserved, hotspot‐driven binding mode (Fig. 1A), the binding affinity and cellular potency of the 1E6 TCR for the different APLs was substantially affected, independently of pHLA stability (Fig. 1B) [19]. Thus, this well‐characterised set of APLs provided a biological relevant model system to further examine the contribution of the antigenic peptide on HLA flexibility.

Molecular flexibility can be usefully thought of as the transitions between different conformational states (energetic minima) on the protein FEL. Combined pressure/temperature (*p*/*T*) denaturation studies have been used in a number of cases to extract the complete suite of thermodynamic parameters that define the FEL for protein folding, so‐called elliptical phase diagrams [54]. In the present study, we wished to explore the FEL specifically relating to native protein conformational change. Nondenaturing hydrostatic pressure is an excellent probe of native protein dynamics since it acts by perturbing the pre‐existing equilibrium of states, favouring more compact conformations [55]. Nondenaturing pressure therefore gives access to the conformational changes that are natively accessible on the proteins FEL.

**Fig. 2:**
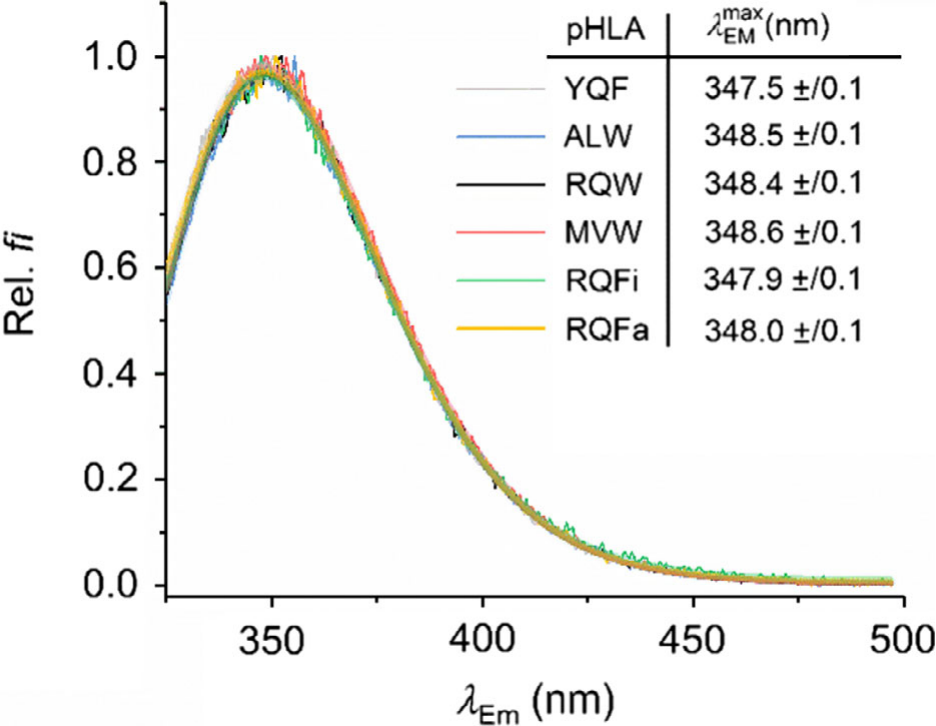
pHLA Trp emission spectra (excited at 295 nm). Solid transparent lines are the fits to Eqn 1. *Inset*, resulting values for $\lambda_{\text{Em}}^{\max}$ extracted from fits to Eqn 1. YQF is shown in grey, ALW is shown in blue, RQW is shown in black, MVW is shown in red, RQFi is shown in green and RQFa is shown in yellow.

Intrinsic Trp emission is a ready reporter of the effect of *p*/*T* perturbation because Trp emission intensity and the structure of Trp emissions spectra are sensitive to changes in the immediate molecular environment through a range of mechanisms [56]. That is, Trp emission spectra can provide accurate metrics of changes in protein tertiary structure. Figure 2 shows the emission spectra for the pHLA complexes used in this study excited at 295 nm. These data show that the structure of the Trp emission spectra is essentially identical, as assessed by the fitting to a skewed Gaussian model, which accurately tracks changes in structure of such spectra,

(1) $$fi=f_{\max}\exp\left(-\ln2\right){\left(\frac{\ln\left(1+\frac{2b\left(\lambda_{\text{Em}}-\lambda_{\text{Em}}^{\max}\right)}{w}\right)}{b}\right)}^{2}$$

where *fi* is the measured fluorescence intensity, *f*_max_ is the maximum emission intensity at wavelength $\lambda_{\text{Em}}^{\max}$, with a full width at half‐maximal of *w,* and the ‘skewness’ is given by *b*. From Fig. 2, fitting to Eqn 1 shows that the emission maxima for each pHLA complex are essentially identical (Fig. 2, *inset*), suggesting that the tertiary structure of the pHLAs is similar and the different peptides have not induced a change in tertiary structure. Moreover, that the $\lambda_{\text{Em}}^{\max}$ values in Fig. 2 are essentially invariant suggests that there is no significant fraction of free HLA, which would otherwise be unfolded and manifest a change in $\lambda_{\text{Em}}^{\max}$.

As above, changes in Trp emission are reporters of protein conformational change and as such can be used to calculate an equilibrium constant for the change across a perturbation series. The change in Trp emission can be converted to an equilibrium constant,

(2) $$\frac{F_{i}}{\sum F}\left(p,\;T\right)=\frac{K\;(p,\;T)}{1+K\;(p,\;T)}$$

where *F*_i_ is the integral of the emission intensity of Trp for a given *p/T*. For a simple 2‐state transition, for example an equilibrium between 2 conformational substates, the temperature dependence of the equilibrium constant is given by,

(3) lnK=(-ΔG)/RT

**Fig. 3:**
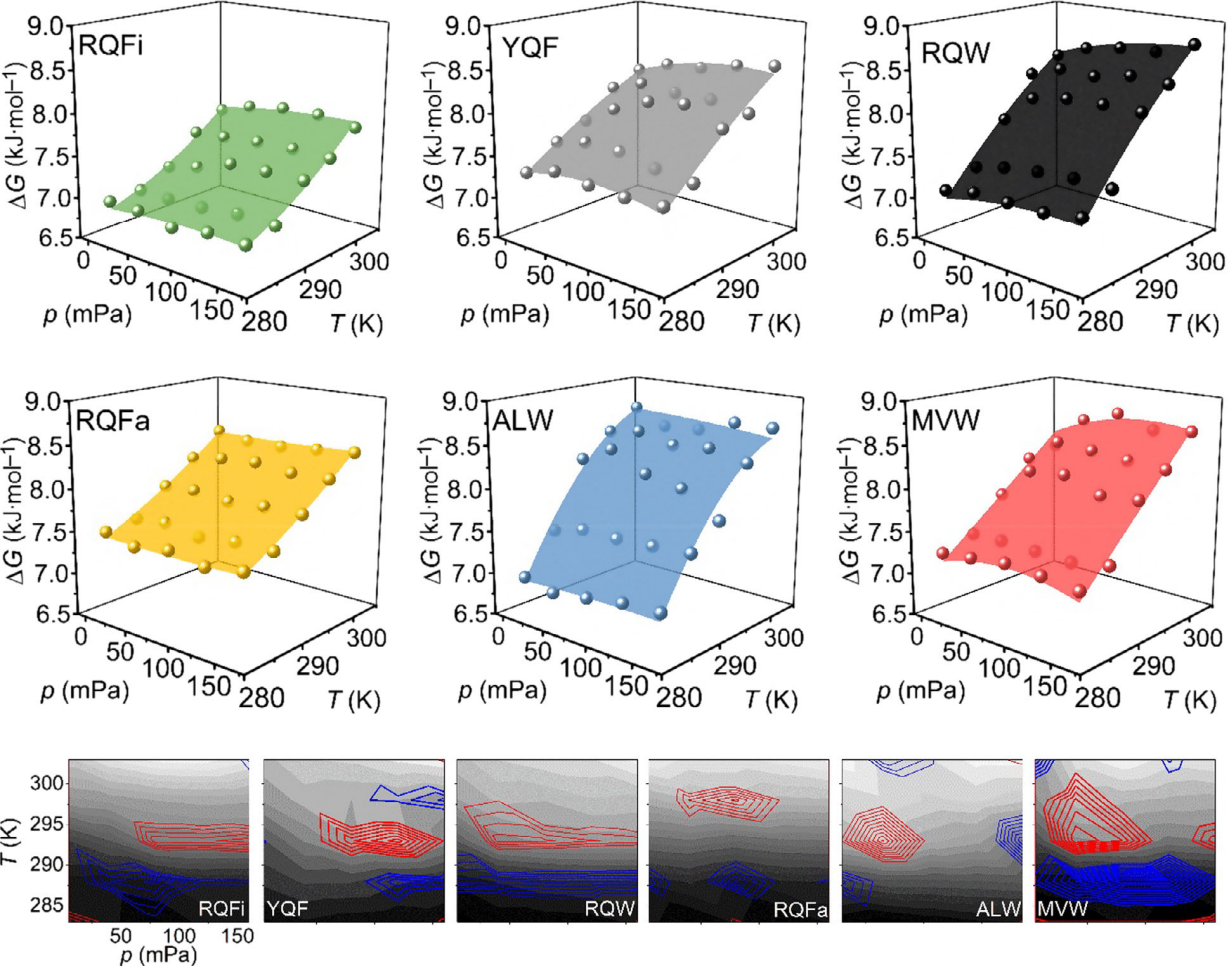
Combined p/T matrices for each pHLA studied. Solid spheres represent the experimental data, transformed to ΔG as Eqn 3. The coloured surfaces are then the resulting fit of these values to Eqn 4. Each panel is labelled as the specific pHLA complex. The grey panels (bottom) show the corresponding residuals for each fit labelled with the associated pHLA complex as the main panels. Red and blue contour lines are the + and − 25% limits, respectively. The concentration of pHLA in each experiment was between 6 and 12 µm (0.3–0.5 mg·mL^−1^), adjusted to give the best signal in the fluorimeter without inducing the inner filter effect.

The combined *p*/*T* dependence of Δ*G* reflects the free energy difference between the 2 notional substates and so is a proxy for the degree of conformational ‘flexibility’. ΔG*_P_*_,_ *_T_* is then given by,

(4) $$\begin{array}{cc} \Delta G_{P,T} & =\Delta G_{0}+\Delta V_{0}\left(P-P_{0}\right)+\Delta \alpha^{\prime }\left(P-P_{0}\right)\left(T-T_{0}\right)+\frac{\Delta \beta^{\prime }}{2}{\left(P-P_{0}\right)}^{2}-\Delta S_{0}\left(T-T_{0}\right)\\ & \;-\Delta C_{P}\left[T\left(\ln\left(\frac{T}{T_{0}}\right)-1\right)+T_{0}\right] \end{array}$$

where *T*_0_ is a reference temperature. Δ*H*, Δ*S*, Δ*G*_0_, Δ*C_p_*, Δ*V*_0_, Δ*β* and Δ*α* reflect the changes in enthalpy, entropy, Gibbs free energy, heat capacity, activation volume, compressibility and expansivity between the 2 notional conformational substates that define the equilibrium, respectively. Note that this model assumes both Δ*C_p_* and Δ*α* are constant with respect to both pressure and temperature. The model further assumes a two‐state transition because the model for a more complex number of states would be intractable when fitting the experimental data. We note that one expects a far larger number of conformational substates to be present, since the FEL is composed of ever more discrete local minima. However, the assumption of a two‐state system provides a means to explore and compare major differences in protein flexibility without overfitting the data. The data in Fig. 3 show a reasonable fit to the Eqn 4 as assessed from the relatively high *R* ^2^ values (all > 0.965) and given the residuals of the fits (Fig. 3; bottom panels). Moreover, none of the fitting parameters were directly dependent on one another as assessed from the variance–covariance matrix. The quality of the fit in each case is reflected in the associated error of the fitted parameters (Table 1).

**Table 1:** Parameters extracted from surface fits shown in Fig. 2. T_m_ values previously published [19]

	*T*_m_ (^o^C)	Δ*G*_0_ (kJ·mol^−1^)	Δ*S* (kJ·mol^−1^)	Δ*C*_p_ (kJ·mol^−1^·K^−1^)	Δ*V* (cm^3^·mol^−1^) × 10^−3^	Δ*β* (cm^3^·mol^−1^·mPa^−1^) × 10^−5^	Δ*α* (K^−1^) × 10^−5^
RQFi	58.8	8.1 ± 0.1	−0.07 ± −0.6	−0.6 ± 0.2	2 ± 1	−1.3 ± 1.1	3.4 ± 3.5
YQF	60.3	8.3 ± 0.1	−0.02 ± 0.33	0.3 ± 0.3	5 ± 1	−3.6 ± 1.5	8.5 ± 4.9
RQW	54.3	8.7 ± 0.2	−0.04 ± 0.02	0.6 ± 0.4	4 ± 2	2.4 ± 2.1	5.0 ± 6.7
RQFa	49.4	8.8 ± 0.1	−0.06 ± 0.01	−0.3 ± 0.2	0 ± 1	6.3 ± 1.0	1.1 ± 3.1
ALW	60	8.7 ± 0.2	−0.01 ± 0.02	1.7 ± 0.3	0 ± 2	−7.1 ± 2.0	−4.6 ± 6.3
MVW	56.7	8.8 ± 0.2	−0.06 ± 0.02	0.0 ± 0.4	6 ± 2	−4.8 ± 2.4	8.7 ± 7.7

These analyses demonstrated that the *p*/*T* relationship clearly differed for different pHLA complexes (Fig. 3, Table 1). The contribution of the different thermodynamic parameters to the magnitude of Δ*G* was highly specific for each peptide sequence. For example, with RQFa and RQFi, the contribution from Δ*S* was large compared with other parameters; for ALW, the contribution from Δ*C_p_* was large compared with other parameters. MVW, RQW and YQF peptides had significant contributions from Δ*V*_0_ and Δ*β*, which were not observed for RQFa, RQFi and ALW peptides. We caution that we have not been able to monitor the concentration of free HLA at different pressures and so our data could conceivably be convolved of some fraction of free HLA. That said, free HLA is known to be extremely unstable [57, 58] and we found our pressure dependence data to be fully reversible, suggesting that if any dissociation occurred on the timescale of the experiment it was not obviously detectable and so by inference, small.

The significant differences in the magnitude of Δ*C_p_* are particularly notable given that a major contribution to Δ*C_p_* for large protein conformational changes and protein folding is the difference in hydration state between the conformational states that are accessed [59]. Similarly, studies with model systems have found that the magnitude and sign of Δα is sensitive to the solvation environment (changes to water structure) [60]. Zero values of Δα imply a ‘rigid’ protein, and positive Δα values are correlated with exposure of hydrophobic residues [61]. Whilst there were some differences outside of error for Δα for the specific pHLAs, we note the large error on these values. That the magnitude of Δ*C_p_* varies significantly (including sign inversions) suggests each pHLA explores conformational substates with unique hydration states, and this implies each pHLA samples unique conformational substates within an equilibrium population. Despite the implied differences in conformational flexibility, the magnitude of Δ*G*_0_ (Table 1) can be similar for the different pHLAs. We interpret these data, taken together, as reflecting that the conformational substates explored by each pHLA are different, but the population distributions across those states are similar.

The p*/T* data point to a peptide sequence‐specific effect on the FEL reflecting HLA conformational flexibility. That is, different peptide sequences affect pHLA molecular flexibility in different ways. Most intriguingly, our data suggest the peptide–HLA interaction is governed by a complex interplay of a range of different thermodynamic contributions, which do not have an obvious relationship to peptide sequence.

### Molecular dynamics simulations identify both local and distal changes in flexibility for different peptide cargos

The *p/T* analysis demonstrated significant differences in the global molecular flexibility and thermodynamics of the pHLA binary complexes, dependent on the peptide cargo. In an attempt to rationalise the differences observed experimentally, we used MD simulations, performing 10 replicas of 150 ns each for each pHLA under investigation, giving a total of 9 µs of simulation time. Using this approach, the (backbone) flexibility of the pHLA can be inferred by calculating the root mean square fluctuations (RMSFs) of each residue's Cα carbon over the course of the simulations.

**Fig. 4:**
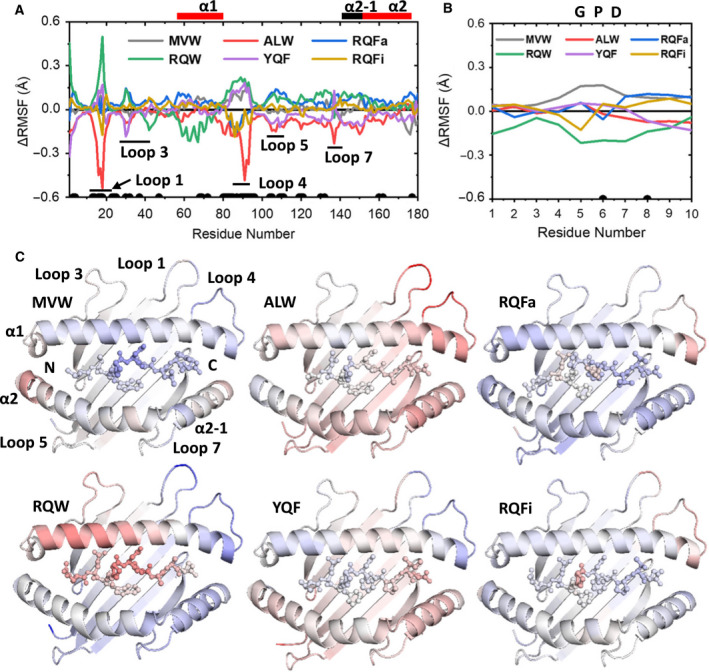
Differences in flexibility in the HLA and the peptide for all 6 pHLA complexes investigated. (A, B) Change in Cα RMSF (average – pHLA) for the α1 and α2 domains (A) and peptide (B), meaning a positive ΔRMSF value indicates an increase in rigidity for that pHLA complex residue relative to the average. The black dots towards the bottom of each graph indicate residues with significantly different ΔRMSF values as determined by a two‐sample *t*‐test (*P* < 0.05). (C) ΔRMSF values as shown in A–B colour mapped on the pHLA structure (HLA as cartoon, peptide as ball and stick), with blue indicating increasing rigidity and red indicating increasing flexibility (again relative to the average RMSF value for that residue). Heat mapping is scaled from –0.5 to 0.5 Å for all complexes. Images were generated with pymol 1.8.6 [88].

**Fig. 5:**
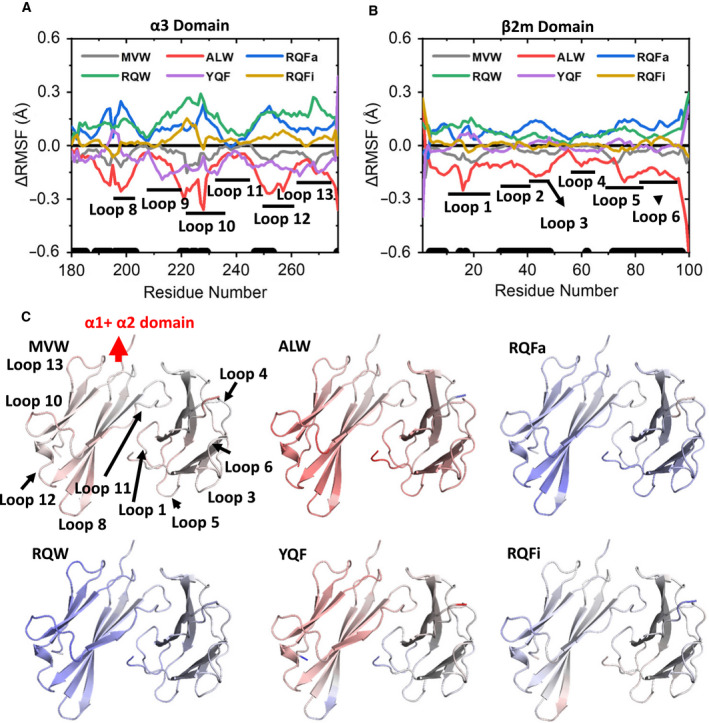
Differences in flexibility in the HLA molecules outside of the peptide‐HLA binding groove for all 6 pHLA complexes investigated. (A, B) Change in Cα RMSF (average – pHLA) for the α3 domain (A) and β2m (B), meaning a positive ΔRMSF value indicates an increase in rigidity for that pHLA complex residue relative to the average. The black dots towards the bottom of each graph indicate residues with significantly different ΔRMSF values as determined by a two‐sample *t*‐test (*P* < 0.05). (C) ΔRMSF values as shown in A–B colour‐mapped on the pHLA structure, with blue indicating increasing rigidity and red indicating increasing flexibility (again relative to the average RMSF value for that residue). Heat mapping is scaled from –0.5 to 0.5 Å for all complexes. Images were generated with pymol 1.8.6 [88].

To investigate the impact of different peptide cargos on the flexibility of the HLA, we first calculated the Cα RMSF for the peptide and the α1 and α2 domains of the pHLA (Fig. 4). We then extended this analysis to the α3 and β2m domains for each pHLA (Fig. 5). As we were primarily interested in the differences in pHLA flexibility with different peptide cargos, we calculated the average RMSF value for each residue in all complexes, and subtracted this from each pHLA complex RMSF value, meaning that a residue with a positive ΔRMSF value indicates an increased flexibility compared with the average. We evaluated the significance of the ΔRMSF differences observed by performing a two‐sample *t*‐test between the most and least rigid pHLA molecules for each position.

Significant differences (*P* < 0.05) were observed for only 2 of the peptide residues (of 10 total). Interestingly, flexibility differences were not necessarily correlated with regions of the peptide that differed between APLs, demonstrating the interconnected nature of the peptide. For instance, the N‐termini had largely very similar flexibility (with the exception of RQW), despite the N‐terminal residues differing substantially between peptides. In contrast, ΔRMSF analysis of the central ‘GPD’ motif of peptide (known to be the main binding site for the TCR and conserved in all APLs investigated in terms of sequence and conformation) demonstrated significant changes in flexibility, particularly between RQW and MVW, which showed increased and decreased flexibility relative to the average, respectively. This is of particular interest as the conserved ‘GPD’ motif accounts for 41‐50% of all contacts between the 1E6 TCR and the 6 pHLA complexes investigated [19]. These changes in peptide flexibility could, therefore, have a direct impact on the interaction between the TCR–pHLA complexes (for instance by modulating the entropic cost of binding).

In contrast to the peptides, which demonstrated relatively small changes in flexibility, significant differences (*P* < 0.05) in flexibility were detected in 45 residues (out of 180 total) for the α1 and α2 helices, 35 residues (out of 97 total) in the α3 domain and 54 residues (out of 100 total) in the β2m domain of the HLA. Whilst it is possible that additional HLA residues show differences in flexibility for different peptide cargos, our results demonstrate the importance of performing many replicas and statistical analysis on those replicas to prevent the observation of what may be false positives [62]. The observed significant differences in flexibility were largely confined to the solvent‐exposed loops in the HLA domains, including loops 3 and 5 in the α1 and α2 domains, respectively. These loops are known to play a role during interactions with tapasin and TAPBPR during peptide editing in the ER [63, 64]. A comparatively smaller number of residues that make up the α1, α2 and α2‐1 helices of the peptide‐binding groove showed significantly different flexibilities. These differences were limited to a central region of the α1‐helix (residues 68–72), the C‐terminal portion of the α1‐helix (residues 80–85) and the middle of the α2‐helix (residues 160–162). Additionally, loops 8 and 10 on the α3 domain and loop 6 on the β2m domain, which are known to play a role in interactions with the CD8 co‐receptor [12], demonstrated high degrees of differences in flexibility. We also note that the statistically significant changes in flexibility we detected in loop 10 in the α3 domain with different peptide cargos are consistent with a previous report demonstrating flexible tuning of this loop during peptide binding [50]. Thus, these changes in flexibility, dependent on peptide cargo, could play a role in tuning the antigen‐processing pathway, or in modulating the interaction with the CD8 co‐receptor, which is known to play a key role in altering T‐cell potency and cross‐reactivity [17, 65, 66, 67]. Overall, differences in flexibility identified by MD analysis were largely observed in the HLA, despite the differences in sequence being confined to the peptide cargo. This unexpected finding of ‘the tail wagging the dog’ may be indicative of allosteric mechanisms in which the sequence of the peptide modulates regions of the HLA known to play a role in different immunological pathways.

#### Peptide‐dependent changes in the binding groove geometry

**Fig. 6:**
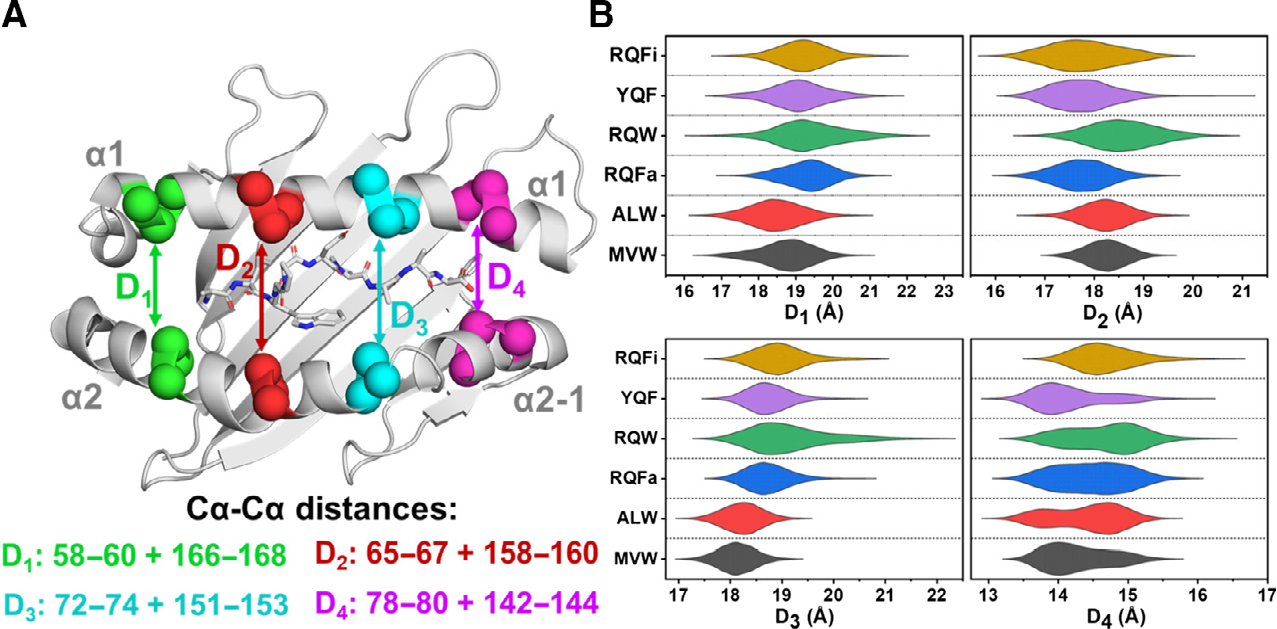
Differences in the HLA‐binding groove widths for all 6 pHLA complexes simulated. (A) Visual representation of the residues selected for each measurement, with an exemplar peptide shown for reference (the peptide N‐terminus is located on the left‐hand side of the figure). For all four measurements, the centre of mass of 3 Cα carbon atoms on each helix is used for the measurement. Images were generated with pymol 1.8.6 [88]. (B) Violin plots based on histograms of the groove widths sampled in MD simulations as labelled in A with RQFi shown in yellow, YQF shown in mauve, RQW shown in green, RQFa shown in blue, ALW shown in red and MVW shown in black. Plots show the distribution of groove widths sampled by the HLA at the four individual measurement points (as shown in A).

Whilst our RMSF calculations on the HLA residues that make up the peptide‐binding site (Fig. 4) identified many of the decorating loops to show significantly different flexibilities dependent on the peptide cargo, differences in the flexibility of HLA residues on the α1, α2 and α2‐1 helices were largely insignificant. This suggests that their (backbone) mobility is highly similar, at least for the peptides studied here. However, whilst these residues may have similar mobilities, they may sample different ranges of conformational space depending on the peptide bound (and thus modulated by the peptide sequence). A peptide‐dependent effect on the groove width (distance between the binding groove helices) has indeed been observed previously in similar systems [49, 50, 68, 69, 70]. Of particular importance may be the peptide‐dependent modulation of the C‐terminal binding site groove widths (located near the break between the α2 and α2‐1 helices); the α2‐1 helix was indicated to ‘swing out’ in order for peptide editing to occur with the chaperones Tapasin or TAPBPR [63, 64, 71, 72]. Comparison of the binding groove widths sampled for each pHLA complex studied here (Fig. 6) shows clear evidence of peptide‐dependent modulation, both in terms of the average binding groove distance and in terms of range of distances sampled. Of particular note, both ALW and MVW sample a much tighter distribution of binding groove widths towards both the N‐terminal and central portions of the peptide (D_1_, D_2_ and D_3_) as compared to RQW. Focussing on sequence‐specific effects, residues with a bulky position 1 residue (RQFa, RQW, RQFi and YQF) tend to sample larger groove widths towards the N‐terminal binding site, as expected (D_1_ in Fig. 6). The size of D_2_ appears to be regulated primarily by position 3 of the peptide, with tryptophan‐containing peptides (MVW, ALW and RQW) sampling larger groove widths as compared to phenylalanine containing peptides (RQFa, YQF and RQFi). For the remaining two groove widths investigated (D_3_ and D_4_), whilst there are clear differences in conformational sampling for the different pHLA complexes, the large differences between the peptide sequences preclude assigning specific effects to specific residues/positions. Interestingly, two different substates in measurement D_4_ are observed for all peptides bar RQFi. These different states relate to the degree to which the α2‐1 helix has swung out from the α1‐helix, and peptide‐dependent regulation of the sampling of these two states is likely important for regulating peptide editing (see Results and Discussion above).

### Identification of correlated motions between the peptide and HLA

**Fig. 7:**
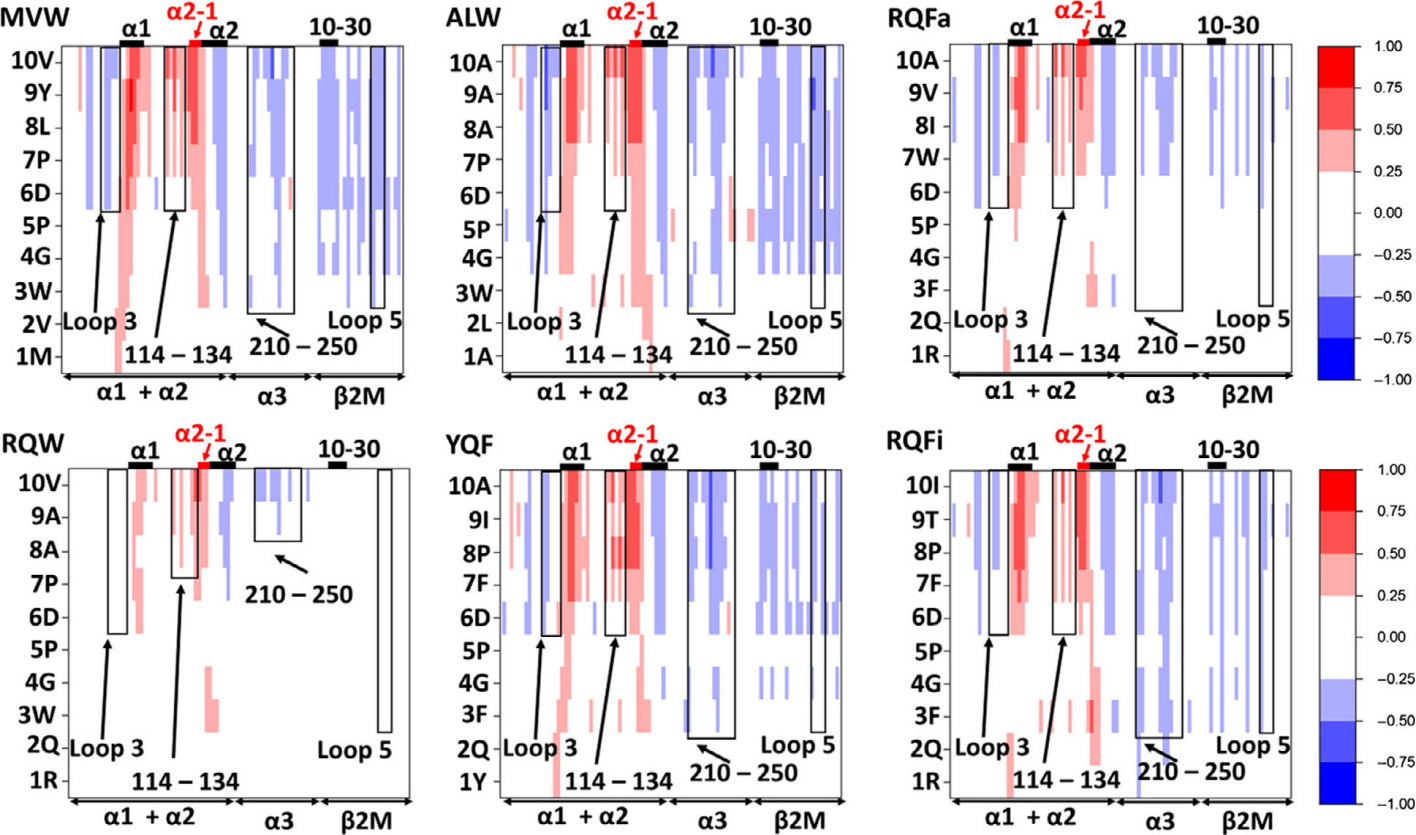
Dynamic cross‐correlation matrices (DCCMs) for all 6 pHLA complexes simulated. On the y‐axis is each residue of the peptide, which is plotted against all other residues (377 total) on the HLA. The matrices are colour‐mapped according to the degree of correlated motion between the two residues, with a value of +1 meaning perfectly correlated and –1 meaning perfectly anticorrelated motion.

With the observed significant differences in flexibility for regions both local and distal from the peptide‐binding site in mind, we computed dynamic cross‐correlation matrices (DCCMs) for all 6 pHLAs under investigation. DCCMs measure the degree of correlated motion between each atom (in this case the Cα carbon of each residue) over the course of the simulation(s). The measurement assigns a value between +1 (perfectly correlated motion) and *–*1 (perfectly anticorrelated motion), with 0 indicating no correlation between the residues. This analysis can therefore be used to identify residues distal from one another that are dynamically linked. To focus primarily on the relationship between the peptide and the HLA, we truncated the obtained DCCMs to allow for easier analysis of their relationship (Fig. 7). Large differences in the overall degree of correlated motion between the peptide and HLA occur, with RQW most strikingly showing a decreased level of correlated motion. Further, a much larger degree of coupling between the C‐terminal end of the peptide and the rest of the HLA is observed, as compared to the N‐terminus and central portion of the peptide. Thus, these data suggest that the C‐terminal residues of the peptide may play a more important role in regulating the global dynamics of the HLA, possibly via the F‐pocket of the HLA‐binding groove. In particular, we observed consistently positively correlated motion between the C‐terminal residues of the peptide and the α1 helix as well as residues 114–134, which make up a large part of the F‐pocket. Interestingly, we also observed a consistent change across all pHLA complexes of positively correlated‐to‐anticorrelated motion along the α2 helix (positive starting at the α2‐1 portion of the helix). The degree of correlation between the peptide and domains distal from the peptide‐binding site (α3 and β2m domains) also showed consistent regions of correlated motions for different pHLA complexes. Whilst in the case of RQW, and to a lesser extent RQFa, these correlations were weaker, and residues within the range 210‐250 on the α3 domain showed correlated motion to the peptide. These residues include those in loop 10, which we herein, and others [50], observed significant differences in flexibility dependent on the peptide cargo.

### Peptide‐dependent tuning of the allosteric communication network

**Fig. 8:**
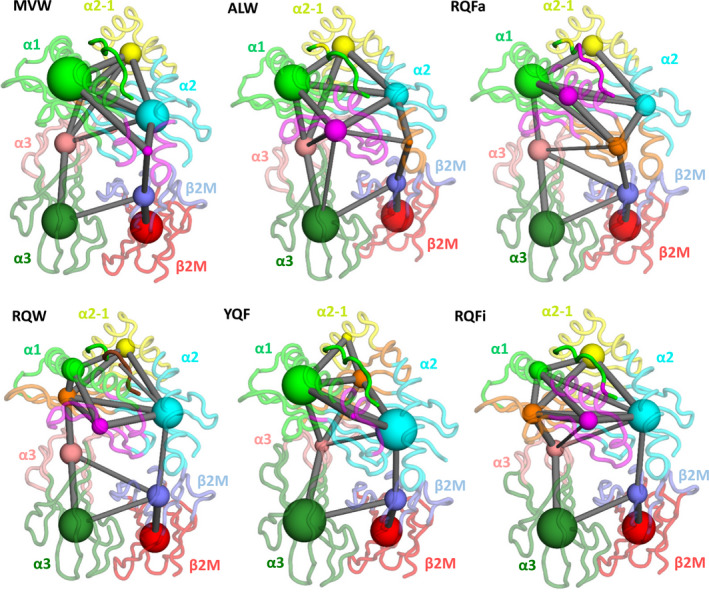
Peptide‐dependent tuning of the allosteric communication network. Community networks determined for all pHLA complexes studied. Networked communities are shown as coloured spheres, with the radii of the sphere indicating the number of residues within the community. Edges between the nodes/communities represent communication pathways between the nodes, with the thickness of the edge indicating the degree of correlation between the two communities (thicker = greater correlation). All pHLA complexes are shown from the same orientation, such that N‐terminus of the peptide is in the foreground.

The observed differences in flexibility and correlated motions for different peptide cargos in the HLA point to an allosterically linked network across the pHLA complex. With this in mind, we turned to community network analysis (CNA) [73] to determine the mechanisms by which the peptide communicates dynamical changes to regions distal from the binding site. In CNA, residues are grouped into ‘communities’ of similar dynamics (communities are groups of residues local to one another that share highly correlated motions). The strength of the communication pathway between different communities is determined by the overall amount of correlated motion between members of the 2 given different communities. These data can therefore be represented in graphical form, in which a node corresponds to a community (with the size of the node indicating the number of residues in that community), and edges between nodes indicating the strength of the communication pathway (with an increased thickness indicating increased correlation) (Fig. 8). The pHLA complexes were partitioned into 9 communities each (chosen based on a consistently high modularity score and an ability to partition the different pHLA complexes as similarly as possible), apart from RQW, which was partitioned into 10 communities because the first 6 residues of the peptide consistently grouped into their own community even at much lower overall community numbers (see Materials and methods for further information). All communities located in the peptide‐binding groove (peptide and α1 and α2 domains) were highly interconnected to one another. Communication from the binding groove to the β2m domain appeared to occur through a single community, generally located towards the end of the α1 helix and part of the residues that form the F‐pocket (peptide C‐terminal binding site). This contrasted with observations made for the top portion of the α3 domain, in which multiple communities in the peptide‐binding groove showed a significant level of directly correlated communication with residues belonging to the α3 domain. These observations may help to rationalise why the C‐terminal peptide residues tended to show a much greater degree of correlated motions with HLA residues. That is, the residues responsible for binding the C‐terminal portion of the peptide appear to be dynamically linked to both the α3 and β2m domains, in contrast to the residues responsible for binding the N‐terminal portion of the peptide.

The above results are supported by a previous study on how different peptides were able to modulate the flexibility of loop 10 in the α3 domain [50]. In this previous study, the major communication pathway found was from the C‐terminal binding site through to the top portion of the β2m domain and then into the α3 domain. This observation compares well with our community network analysis across all complexes studied here, revealing a highly similar communication pathway from the C‐terminal binding site to the α3 domain (from the cyan to the dark blue and then to dark green communities in Fig. 8).

## Conclusions

Here, we used cutting edge experimental approaches and molecular dynamics simulations to demonstrate that the peptide cargo is able to tune the conformational dynamics of HLA. More specifically, the precise amino acid composition of the peptide cargo differentially engages a network of correlated protein dynamics that spans the HLA. For instance, the C‐terminus of the peptide appears to be able to regulate the conformational dynamics of the entire pHLA complex as well as the main TCR–peptide contact zone, potentially modulating TCR binding. Our data point to the peptide cargo having the ability to tune a network of allosteric dynamics in the pHLA complex and may play a role in tuning a number of pathways involved in T‐cell‐mediated immunity. These include peptide editing during antigen processing, interactions with the CD8 co‐receptor, and direct TCR interactions with the peptide and HLA. These findings may be pertinent for peptide vaccine design and may help explain why even minor alterations in peptide sequence can completely alter the direction of the immune response [44, 74, 75]. Our study also has broader implications for the understanding of protein interaction networks, particularly allosteric mechanisms, in which changes in a relatively small component of the protein complex (in this case a few mutations in a 10‐amino acid peptide) can a modulate flexibility distal to the changes and throughout the complex (in this case, HLA, a 4‐domain protein complex, made up of nearly 400 amino acids).

## Materials and methods

### Protein expression and refolding

HLA‐A*02:01 and β2m were expressed and refolded using competent BL21 DE3 E. coli cells transfected with pGMT7 expression plasmids as previously described [76, 77]. Refolded protein was purified by anion exchange using a Poros 50HQ column, followed by size exclusion into phosphate‐buffered saline using gel filtration column – Superdex™ 200 Increase 10/300 GL. Purification followed a previously described protocol [76, 77]. Dynamic light scattering (Zetasizer, Malvern Products, Malvern, UK) was used to confirm sample homogeneity. For pressure/temperature varying fluorescence measurements, samples were exchanged into a HEPES buffer (50 mm HEPES, 150 mm NaCl, pH 7.4) using a PD‐10 desalting column containing Sephadex™ G‐25 medium, following manufacturer instructions. We note that the refolding of HLA, in the presence of peptide, with subsequent purification, excludes uncomplexed pHLA.

### Pressure/temperature‐dependent fluorimetry

Pressure/temperature measurements were performed using an ISS high‐pressure cell (ISS, Champaign, IL, USA) fitted with a custom fibre optic mounting connecting to the fluorimeter and the water bath. Peptide–HLA complexes were excited at 295 nm, and tryptophan emission was measured between 325 and 500 nm. Emission and excitation slits were set to 15 nm to minimise the signal‐to‐noise ratio (due to optimal setup of the pressure cell). Initial measurements were made at 10 °C and increased in 5 °C increments up to 30 °C. The pressure dependence at each temperature was measured at 50, 400, 800, 1200 and 1600 bar. Measurements were taken in triplicate. Following each full pressure/temperature range, repeat scans were taken at lower pressure/temperature conditions to ensure extreme pressure/temperature conditions had not denatured the protein. For all measurements, the appropriate buffer controls were subtracted prior to data processing. Fitting Eqn 4 to the data gave typical *R* ^2^ values > 0.965. Fitting was achieved using originpro (OriginLab). The concentration of pHLA in each experiment was between 6 µm and 12 µm (0.3–0.5 mg·mL^−1^), adjusted to give the best signal in the fluorimeter without inducing the inner filter effect. The values given in Table 1 are the result of three experimental replicates. The data are fit to Eqn 4 accounting for the standard deviation of each data point. The resulting parameters extracted from the data (Table 1) show the error calculated from the fit.

### MD simulations

Previously solved X‐ray crystal structures of the 6 pHLA complexes (PDB I.D.s: 3UTQ, 5C0E, 5C0F, 5C0H, 5C0I and 5C0J) [2, 19] were used as the starting point for all MD simulations. Any missing residues were added using modeller v9 [78]. propka 3.0 [79] was used to predict the protonation states of all proteins investigated at pH 7 (resulting in all residues being simulated in their standard protonation states). MolProbity [80] was used to determine the optimum tautomerisation states for every His residue and make any required Asn/Gln side‐chain flips (under the criteria of optimising the hydrogen bonding network). The results were visually inspected, and care was taken to ensure consistency between all pHLAs investigated. Histidine residues 4, 71, 115, 189, 193, 261 (Chain A) and 51 (Chain B) were simulated as singly protonated on their Nδ1 nitrogen, with all other histidine residues simulated as singly protonated on their Nε2 nitrogen. All systems were then solvated in an octahedral water box (retaining any crystal waters) such that no protein atom was within 10 Å of the box boundary. Simulations were performed at an effective [NaCl] of 150 mm (to match experiments), with excess Na+ ions added as required to ensure neutrality. MD simulations were performed using Amber16, describing the protein and water molecules with the ff14SB force field [81] and TIP3P water model [82], respectively. Following a protocol of minimisation, heating and equilibration (see section ‘Structure equilibration procedure’ below), all pHLA complexes were subjected to 10 × 150 ns of production MD simulations in the NPT ensemble (at 300 K and 1 atm), with snapshots collected every 10 ps. Production MD simulations were performed using a 2 fs time step and with the SHAKE algorithm applied. An 8 Å direct space nonbonded cut‐off was applied with long‐range electrostatics evaluated using the particle mesh Ewald algorithm [83]. Temperature was regulated using Langevin temperature control (collision frequency of 1 ps^−1^), whilst pressure was controlled with a Berendsen barostat (setting the pressure relaxation time to 1 ps).

### MD trajectory analysis

Routine trajectory analysis was performed with CPPTRAJ [84]. Cα RMSF calculations were performed for all complexes after discarding the first 10 ns of simulation time (for equilibration) and averaged over each run. RMS fitting was performed to the Cα of stable (over the course of our MD simulations) secondary structure residues of the HLA. We used the following residues for RMS fitting: 4–13, 22–38, 51–54, 58–86, 95–104, 111–127, 134–181, 187–196, 199–209, 215–220 and 242–263 of Chain A (i.e. the α1, α2 and α3 domains), and residues 6–11, 21–30, 36–41, 60–70, 78–83 and 91–94 of Chain B (i.e. the β2m domain). For RMSF, DCCM and CNA calculations, RMS fitting was first performed to the crystal structure in order to create an average structure. Following this, all snapshots were then refitted to the average structure for the subsequent calculations. DCCMs and CNA were calculated using a combination of the Bio3D [85] and igraph [86] libraries within the package *R*. Briefly, all 10 independent simulations were combined into a single trajectory, RMS fitting each frame to an average structure of all ten simulations combined. DCCMs were calculated for all 387 × 387 residues in each pHLA before truncating the matrix to show the degree of correlated motion between the peptide and all HLA residues. CNA was performed on the aforementioned complete DCCM results, using a Girvan–Newman clustering protocol [73] to cluster communities of similar dynamics together. Edges with a correlation score of < |0.4| were discarded prior to clustering. The resulting communities were further filtered using a maximum distance cut‐off between pairs of atoms of 8 Å (throughout 100% of the simulation time). Whilst the standard procedure in CNA is to plot the community number that gives the highest modularity, it is also acceptable when comparing multiple similar complexes, to choose a high scoring modulatory value that better groups the resulting structures [87]. The modularity is a measure of the level of interconnectedness between community members, and the level of correlation with noncommunity members, with a higher score indicating increased intercommunity correlation and decreased intracommunity correlation, and therefore a better division of the data. We choose a community number of 10 for RQW and 9 for all other pHLA complexes based on the above criteria, and in all cases, the difference between the maximal possible modularity score and the selected community score was no greater than 0.02. A value of 10 was selected for RQW as the N‐terminal portion of the peptide consistently grouped to itself even at much lower community numbers (lowest evaluated community number was 4).

### Structure equilibration procedure

Upon preparation of all six pHLA complexes investigated, the following procedure was used to equilibrate structures for production MD simulations performed at 300 K and 1 atm: minimisation of all hydrogen atoms and solvent molecules (including Na^+^ and Cl^−^), using 500 steps of steepest descent followed by 500 steps of conjugate gradient. To keep all other atoms (i.e. the protein heavy atoms) in place during the minimisation, 10 kcal·mol^−1^·Å^−1^ positional restraints were applied. Retaining the positional restraints on all protein heavy atoms, the system was then heated rapidly from 50 to 300 K in the NVT ensemble over the course of 200 ps. This system was again minimised for a further 500 steps of steepest descent followed by 500 steps of conjugate gradient, this time only applying positional restraints (of 5 kcal·mol^−1^·Å^−1^) to the Cα carbon atoms. These Cα restraints were retained as the system was again heated from 25 to 300 K over the course of 50 ps in the NVT ensemble. Simulations were then performed in the NPT ensemble (1 atm, 300 K), first gradually reducing the 5 kcal·mol^−1^·Å^−1^ Cα carbon restraints over the course of 50 ps of simulation time. This was done in 5 steps (5, 4, 3, 2, 1 kcal·mol^−1^·Å^−1^) of 10 ps each. A final 1 ns simulation was then performed in which no restraints were used. The end structure from this run was then used as the starting structure for a production MD simulation. All dynamics steps used the SHAKE algorithm. Simulations performed in the NVT ensemble used Langevin temperature control (with a collision frequency of 1 ps^−1^) and used a simulation time step of 1 fs. Simulations performed in the NPT ensemble again used Langevin temperature control (collision frequency of 1 ps^−1^) and a Berendsen barostat (1 ps pressure relaxation time), with a simulation time step of 2 fs. Simulations of replicas were performed by taking the structures after the second minimisation step (and before the second heating step). Replicas were therefore assigned different random velocity vectors on the subsequent heating step.

## Author contributions

JRH, DAMC, DKC and CRP performed and/or directed experiments. RMC and MWvdK performed and/or directed simulations. JRH, RMC, DAMC, AKS, VLA, MWvdK, DKC and CRP analysed data and critiqued the manuscript. MWvdK, DKC and CRP conceived and directed the project. JRH, RMC, MWvdK, DKC and CRP wrote the manuscript.

## Conflict of interest

DKC is an employee of Immunocore LTD. The authors declare that the research was conducted in the absence of any other commercial or financial relationships that could be construed as a potential conflict of interest.
